# Polyphenol Intervention Ameliorates Non-Alcoholic Fatty Liver Disease: An Updated Comprehensive Systematic Review

**DOI:** 10.3390/nu16234150

**Published:** 2024-11-29

**Authors:** Yazan Ranneh, Alaa S. Bedir, Abdelghafar M. Abu-Elsaoud, Seham Al Raish

**Affiliations:** 1 Department of Nutrition and Dietetics, College of Pharmacy, Al-Ain University, Al-Ain P.O. Box 64141, United Arab Emirates; yazan.ranneh@aau.ac.ae; 2 Department of Nutrition, College of Medicine and Health Science, United Arab Emirates University, Al Ain 15551, United Arab Emirates; 201950078@uaeu.ac.ae; 3 Department of Biology, College of Science, Imam Mohammad Ibn Saud Islamic University (IMSIU), Riyadh 11623, Saudi Arabia; amsmohamed@imamu.edu.sa; 4 Department of Biology, College of Science, United Arab Emirates University, Al-Ain 15551, United Arab Emirates

**Keywords:** fatty liver, inflammation, non-alcoholic, polyphenols, systematic review

## Abstract

Non-alcoholic fatty liver disease (NAFLD) has recently emerged as a challenging metabolic disorder with a strong emphasis on its prevention and management. Polyphenols, a group of naturally occurring plant compounds, have been associated with a decreased risk of various metabolic disorders related to NAFLD. The current systematic review aims to critically assess evidence about the ameliorative effect of polyphenol supplementation on NAFLD patients. A PRISMA systematic search appraisal was conducted in PubMed, Scopus, Web of Science Core Collection, and all relevant studies published prior to April 2024 and met the inclusion criteria were included. Twenty-nine randomized clinical trials (RCTs) comprised 1840 NAFLD patients. The studies primarily examined eleven phenolic compounds, including turmeric, curcumin, resveratrol, genistein, catechin, green tea extract, hesperidin, and silymarin. Turmeric and curcumin decreased liver enzymes, inflammatory cytokines, lipid profile, insulin resistance, and NAFLD score, while resveratrol did not present consistent results across all the studies. Most studies on silymarin showed a reduction in liver enzymes and lipid profile; however, no changes were observed in inflammatory cytokine levels. The dietary supplementation of hesperidin and naringenin or green tea extract caused improvements in liver enzyme, lipid profile, and inflammatory cytokine, while genistein supplementation did not modulate blood lipid profile. In conclusion, dietary supplementation of polyphenols could potentially prevent and ameliorate NAFLD. Still, the inconsistent results across the included RCTs require further clinical research to establish optimal dosage and duration.

## 1. Introduction

Concomitant with the worldwide surge in metabolic syndrome, diabetes, and obesity, an abrupt surge has been witnessed in the ubiquity of non-alcoholic fatty liver disease (NAFLD) [1]. NAFLD exhibits a worldwide prevalence of 30.05%, with the most elevated rates occurring in Latin America (44.37%), followed by Middle East and North Africa (36.53%), South Asia (33.83%), Southeast Asia (33.07%), North America (31.20%) and Western Europe (25.10%) [2]. It is noteworthy that an estimated 22.5% of individuals diagnosed with this non-alcoholic steatohepatitis (NASH) progress to hepatocellular carcinoma, while approximately 20% experience the development of cirrhosis [3].

NAFLD includes a spectrum of clinical and pathological liver conditions, ranging from isolated fat accumulation and fibrosis to cirrhosis, with a potential link to the development of hepatocellular carcinoma. It is characterized by the presence of steatosis in more than 5% of hepatocytes, as detected through histological or radiological examination; NAFLD diagnosis requires the exclusion of secondary factors, such as substantial alcohol consumption (less than 30 g/day for men and less than 20 g/day for women), hereditary liver disorders, or viral hepatitis [4,5,6]. The primary theory explaining the development of NAFLD is known as the “two-hit” hypothesis. According to this model, the first event, or “hit”, results in steatosis, while the second “hit” leads to necro-inflammation and fibrosis. The initial “hit” is often linked to insulin resistance, and various factors such as oxidative stress, cytokine effects, and fatty acid toxicity are considered potential contributors to subsequent hepatocellular injury [5]. Tumor necrosis factor-alpha (TNF-α) in the liver is associated with oxidative stress and may play a role in insulin resistance. Moreover, TNF-α is implicated in fibrosis through the direct activation of hepatic stellate cells and by stimulating the production of tumor growth factor (TGF)-beta, a potent cytokine promoting fibrosis [7]. According to the “second hit” theory, the dysfunction of peripheral adipose tissue in insulin resistance triggers lipid breakdown, elevating free fatty acid levels in the bloodstream. Unhealthy lifestyle choices exacerbate this elevation, causing an overflow of fatty acids to the liver beyond its transport capacity, leading to liver deposition and subsequent steatosis, which is named “the first blow”. Prolonged excessive fat accumulation induces endoplasmic reticulum stress, mitochondrial dysfunction, and oxidative stress. This cascade results in the release of inflammatory factors, intensifying liver cell damage and propelling the shift from simple fatty liver to NASH, rendering it “the second blow”. Peripheral adipose tissue exhibiting insulin resistance diminishes adiponectin secretion while increasing pro-inflammatory factors, amplifying the body’s inflammatory response and exacerbating insulin resistance, establishing a detrimental cycle [8].

In the past 20 years, there has been an increase in NAFLD and NASH not only in Western countries, where a sedentary lifestyle and obesity are prevalent along with high-calorie diets, but also in urban areas of developing countries [9]. Despite several therapeutic options proposed for NAFLD and NASH in recent years, none have proven sufficient in improving liver function and addressing hepatocyte stress and inflammation. Currently, there are no approved treatments for NAFLD, and available options mainly focus on advising lifestyle changes, such as adopting a healthy diet low in fats and carbohydrates and engaging in regular physical activity [10]. One of the most common types of healthy diet is the Mediterranean diet, where polyphenols are abundantly available [10].

Plant-derived foods exhibit a diverse array of secondary metabolites, with polyphenols emerging as a particularly abundant and nutritionally pivotal class of phytochemicals [11]. The collection of phenolic structures exceeds 8000 compounds, with more than 500 identified in plant foods, constituting the dietary polyphenols. In recent decades, many investigations have investigated the health-promoting attributes inherent to polyphenols. The consistent inclusion of polyphenol-rich foods in dietary regimens has demonstrated an association with a potential decrement in the prevalence of conditions such as cardiovascular diseases, colon cancer, liver disorders, obesity, and diabetes [12]. Evidence supports that polyphenols can alleviate oxidative stress and inflammatory cascades, modulate immune responses, and elicit various pharmacological effects [13]. Different in vitro and in vivo studies have found that polyphenols could have a direct impact in alleviating or preventing the onset of NAFLD and NASH by regulating the Nrf-2 and AMPK [11].

Numerous randomized controlled trials (RCTs) investigating the effect of dietary polyphenols on the mitigation of NAFLD or NASH have been documented. Nevertheless, these RCTs’ outcomes and therapeutic interventions exhibit heterogeneity in study populations, polyphenol type, dosages and duration, which makes cross-study comparisons challenging and provides inadequate foundational framework for clinicians in planning treatment protocols for NAFLD or NASH. In addition, a lack of standardized dosage recommendations and consensus on specific phenolic compounds further contributes to inconsistent outcomes. Thus, the current systematic review aims to synthesize data from divers RCTs to clarify common findings, identify trends associated with specific polyphenols, and examine dosage ranges and treatment durations to highlight emerging therapeutic standards that can improve clinical management of NAFLD.

## 2. Materials and Methods

### 2.1. Search Strategy

**Figure 1:**
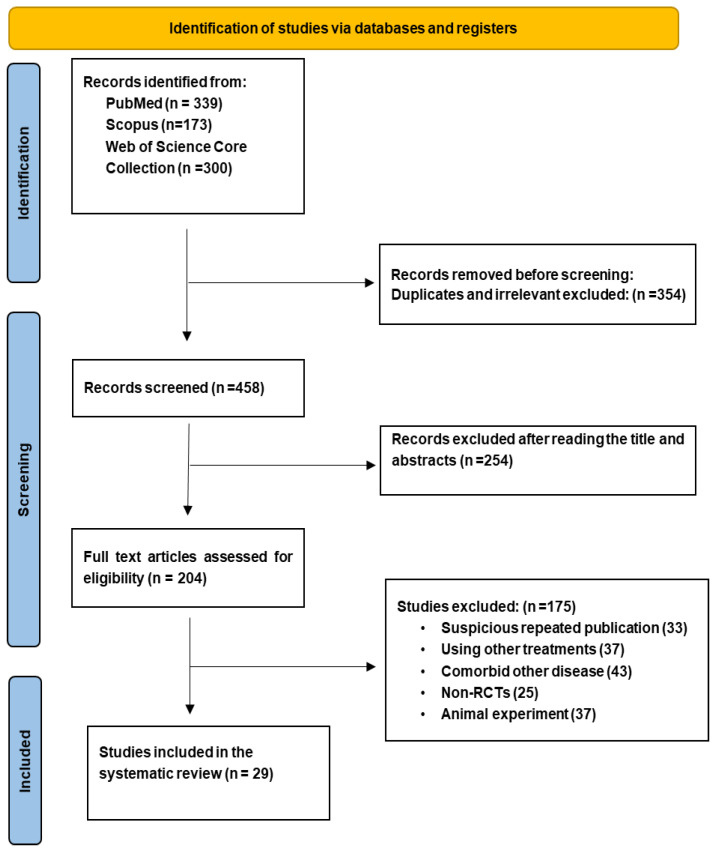
PRISMA flowchart of the included studies in the systematic review.

To ensure transparency and methodological accuracy in the review process, this systematic review aimed to provide a comprehensive and unbiased evaluation of the available evidence by adhering to the PRISMA guidelines and checklist. To identify relevant studies, an electronic literature search was performed in three scientific databases (PubMed, Scopus, and Web of Science Core Collection), and all relevant studies published prior to April 2024 that met the inclusion criteria were included. The search method employed a mix of appropriate keywords and Medical Subject Headings (MeSH) terms related to non-alcoholic fatty liver (NAFLD), non-alcoholic steatohepatitis (NASH), dietary supplement, polyphenols, and intervention (Table S1). Restrictions were applied based on language and publication type. Furthermore, the reference lists of relevant articles and review papers were screened to identify any supplementary eligible studies (Figure 1).

### 2.2. Eligibility Criteria and Study Selection

The following inclusion criteria were established to ensure the selection of suitable studies. The study design included randomized controlled trials (RCTs), case–control studies, cross-sectional studies, and prospective cohort studies. Reviews, conference abstracts, animal studies, and in vitro studies were excluded. The included participants were aged 18 years old or older and diagnosed with NAFLD. Studies examining the effect of polyphenols as a dietary intervention in managing NAFLD were considered for inclusion. The outcomes of interest were critical indicators of NAFLD, including body mass index (BMI), Homeostasis Model Assessment-Insulin Resistance (HOMA-IR), liver enzymes (GGT, ALT, and AST), blood lipids [total cholesterol (TC), TG, high-density lipoprotein cholesterol (HDL-C), low-density lipoprotein cholesterol (LDL-C)], and inflammatory biomarkers. Our systematic review exclusively included articles published in the English language. Initial screening of all identified studies based on titles and abstracts was conducted by two independent reviewers, S.L.P and I.A. Subsequently, full-text articles of included studies were obtained for a more thorough assessment. Any disagreement was addressed through discussion and, when required, with the engagement of a third reviewer to reach a consensus.

### 2.3. Data Extraction and Risk of Bias Assessment

Two independent reviewers conducted data extraction utilizing a standardized form to ensure consistency. Extracted information included the study characteristics such as authorship, publication year, geographical location, study design, sample size, participant demographics, specifics of the dietary intervention, study duration, primary and secondary outcomes, and significant findings. The methodological quality of the included RCTs was evaluated by employing the Cochrane Collaboration’s risk of bias assessment tool. Two reviewers autonomously assessed bias risk, with any disparities resolved through consensus discussions.

## 3. Results

### 3.1. Study Selection and Characteristics

The PRISMA guidelines were followed in reporting the selection process of the included studies, as shown in Figure 1. After performing an initial search, 812 papers were collected, followed by an exclusion of 354 studies due to being duplicates and being irrelevant to the topic. Then, an extensive reading of the titles and abstracts was performed to exclude 254 studies unrelated to polyphenol supplementation and NAFLD. A total of 204 studies were assessed entirely in accordance with the eligibility criteria. Of these, 29 studies were deemed suitable for inclusion in the systematic review [14,15,16,17,18,19,20,21,22,23,24,25,26,27,28,29,30,31,32,33,34,35,36,37,38,39,40,41,42]. 

The main characteristics of the 29 included studies are exhibited in Table 1. Among those, an RCT protocol was presented [29]. In 13 studies, a randomized controlled trial design was employed, while 15 studies utilized a double-blind placebo-controlled trial design where participants and researchers were unaware of the treatment allocation. However, two studies incorporated more than two groups to assess different doses or combinations of treatments [34,39]. The most significant number of trials was executed in Iran, with 20 studies. Following closely behind were studies conducted in Italy, with three trials, and Denmark, with two trials. Additionally, one trial was conducted in Malaysia, China, Australia, and Pakistan. The studies primarily investigated eleven phenolic compounds, including turmeric, curcumin, resveratrol, anthocyanins, naringenin, genistein, catechin, green tea extract, hesperidin, silybin, and silymarin.

As shown in Table 1, 1840 patients with NAFLD were included in the study, and their ages ranged from 18 to 70 years. The sample sizes across the studies varied from a low of 10 subjects per group [25] to a high of 69 subjects per group [37]. Although certain studies did not provide specific gender data, the overall gender distribution across the included studies was approximately 491 males and 429 females. The included trials examined a broad range of phenolic compounds, administered in a single form or combined with other compounds such as vitamin D, E, piperine, phosphatidylcholine, or flaxseed. All phenolic supplements were provided orally in capsule form, ranging from 8 to 48 weeks. Turmeric and its by-product, namely curcumin, was the most examined phenolic compound in 11 studies, while resveratrol and silymarin were studied in six trials for each one.

### 3.2. Liver Enzymes

The primary outcomes of the included studies were liver enzymes (AST, ALP, and ALT), lipid profile, inflammatory cytokines, insulin resistance, and anthropometric changes (Table 1). A total of 28 studies measured liver enzymes, including ALT, AST, and GGT. Curcumin and its derivatives significantly reduced liver enzymes across the included trials [14,15,16,17,18,19,20,21,22]. While resveratrol showed a significant reduction in AST and ALT in three trials [23,24,27], one trial revealed an increase in liver enzymes [25], and two trials failed to show a statistically significant impact [26,28]. Naringenin, catechine, and catechine-rich green tea extract were also found to stimulate a reduction in liver enzymes [30,31,32,33]. Hesperidin supplementation demonstrated a considerable decrease in ALT and GGT but not AST [34,35]. Out of seven trials that used silybin and silymarin supplementation, five trials only showed a significant reduction over AST, ALT, and GGT [36,37,40,41,42].

### 3.3. Lipid Profile

A total of 14 trials reported blood lipid profiles, including TG, LDL, TC, and HDL, while six studies reported specific lipid parameters. Of these 14 trials, seven studies showed significant improvement in blood lipid profile due to turmeric, curcumin, green tea extract, hesperidin, and silymarin [14,15,16,20,33,34,42]. The remaining seven studies involving the consumption of resveratrol [23,25,26], genistein [31], silybin [36], and silymarin [38,40] discovered non-significant amelioration for the whole lipid profile.

### 3.4. Inflammatory Markers and NAFLD Score

Inflammatory markers were assessed in 11 studies with controversial outcomes. In particular, five studies highlighted a significant reduction in TNF-α levels after supplementation with curcumin [17], resveratrol [24], genistein [31], and hesperidin [34,35]. In contrast, three studies on resveratrol [23,25,27] and one study on silybin [36] did not discover any significant improvement in TNF-α levels. Similarly, resveratrol and silybin failed to improve the serum levels of CRP [25,27,36], while green tea extract and hesperidin induced a significant amelioration, as shown in Table 1 [34,35,40]. IL-6 levels were reported to be controversial post-supplementation with resveratrol [25,27,28], but genistein supplementation significantly reduced IL-6 [31].

Seven studies reported NAFLD scores among all the included trials. Five trials found a significant improvement in NAFLD scores due to intervention with silymarin [38], silybin [37], naringenin [30], and curcumin [17,21]. In addition, hepatic fibrosis was noticed to improve after supplementation with curcumin [20], hesperidin [34,35], and silymarin [38].

### 3.5. HOMA-IR and Body Mass Index

A total of nine trials found a significant improvement in HOMA-IR values. This significant modulation was achieved following intervention with turmeric [14], curcumin [18,20], resveratrol [24], genistein [31], green tea extract [33], hesperidin [34], and silybin [36,37]. Also, BMI was highlighted in 16 studies, but in 9 trials, BMI was significantly decreased following intervention with curcumin and turmeric [14,16,20], resveratrol [26], naringenin [30], genistein [31], green tea extract [33], hesperidin [34], and silymarin [42].

### 3.6. Risk of Bias Assessment

**Figure 2:**
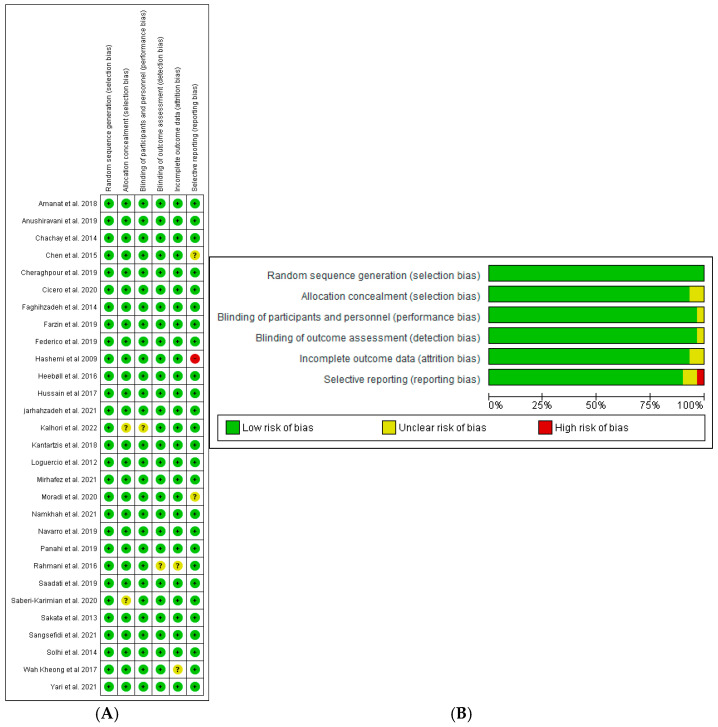
Risk of bias assessment for the included studies. (**A**) summary of risk of bias, (**B**) risk of bias graph. Symbols: (+) low risk, (?) unclear risk of bias, (−) high risk of bias [14,15,16,17,18,19,20,21,22,23,24,25,26,27,28,29,30,31,32,33,34,35,36,37,38,39,40,41,42].

As shown in Figure 2, the risk of bias assessment for the included studies indicated a generally low level of bias across all the domains. In all the included studies, randomization processes were consistently rated as low-risk, indicating cohesive methodologies for participant allocation. The majority of included trials received a low-risk rating in terms of deviation. However, a moderate risk of bias was notable in two studies [14,17]. A total of 28 studies received a low risk of bias in missing outcomes, except for [22]. The domain of outcome measurement was predominantly rated as low-risk, but Kheong et al. (2017) [38] was rated with a moderate risk, raising concern about the consistency of data collection. Finally, the assessment of reported results demonstrated a low risk of bias; however, four studies indicated a moderate risk.

## 4. Discussion

The findings of the current systematic review highlight the potential therapeutic benefits of dietary polyphenols in managing NAFLD. Among the 29 included RCTs involving 1840 participants, curcumin, silymarin, and hesperidin consistently demonstrated significant improvements in liver enzymes (AST, ALT, and GGT), lipid profile (LDL, HDL, TG and TC), and reductions in inflammatory cytokines (TNF-α, IL-6 and CRP). However, other polyphenols including resveratrol showed inconsistent results across trials while naringenin and genistein were supported by a limited number of studies.

The precise prevalence of NAFLD is not known, but it is increasing and in parallel with diseases such as diabetes, dyslipidemia, and obesity. It is believed that the prevalence is 75% among obese patients and can rise to 90% in severely obese patients [43]. NAFLD affects 20% to 30% of the whole world population, and less than 25% are diagnosed with NAFLD in Asian countries. Obesity is a significant factor in the development of NAFLD through genetic predisposition as well as dietary behavior [44]. There is no pharmacological intervention for NAFLD except lifestyle modification with a specific diet and increasing physical activity [11]. Therefore, discovering potential therapeutic agents is still required.

Medicinal plants and their extracted phytochemicals, including polyphenols, have been proposed to ameliorate various diseases for centuries. Polyphenols are a wide range of plant-derived compounds with water-soluble characteristics [45]. However, bioavailability of polyphenols varies widely depending on their chemical structure and forms of delivery [12]. Encapsulation technologies such as liposomal or nanoparticle systems enhance polyphenol absorption and increase systemic availability. For example, nano-formulations including liposomes and polymeric nanoparticles enhance significantly the bioavailability of curcumin by increasing its stability, solubility and resistance to gastrointestinal degradation [46]. Three independent reviews have highlighted that nano-curcumin is effective in reducing liver enzymes, hepatic steatosis, and inflammation [47]. However, a clinical trial investigating the supplementation effect of raw turmeric on liver enzymes and fatty liver degree in NAFLD patients reported no significant changes [48]. Consuming raw dietary polyphenols often exhibited lower bioavailability due to their glycosylated forms, which subsequently reduce the therapeutic effect [46].

In the human diet, polyphenols, like vitamins, are the most abundant antioxidant compounds with many biological activities [49]. The ability of polyphenols to prevent oxidative stress, increase fatty acid beta-oxidation, and regulate insulin resistance has been documented in experimental studies [50]. Furthermore, fruit-rich polyphenols can facilitate the export of hepatic TG and reduce lipogenesis through the brain vagus–liver axis [51]. The hepato-protective properties of certain pure polyphenols, including turmeric, resveratrol, silybin, and catechin, against steatosis have been observed in HegG2 cells challenged with oleic acid [52]. Several RCTs have recently examined phenolic compounds’ efficacy in NAFLD patients. Therefore, the current systematic review aimed to summarize and provide up-to-date clinical evidence by which scientists and clinicians could learn the potential therapeutic possibilities of phenolic isolated compounds. The analysis of 29 RCTs with 1840 patients revealed a promising role of phenolic compounds in ameliorating various biomarkers and clinical outcomes associated with NAFLD.

Liver enzymes were a consistent finding across the included studies, particularly ALT, AST, and GGT. These enzymes mainly involve hepatocellular biological functions, including amino acid metabolism and glutathione synthesis [53]. At the preclinical level, polyphenol supplementations were proven to induce hepatocellular protection, decreasing AST, ALT, and GGT [11]. Most of the included trials indicated low levels of liver enzymes post-phenolic intervention, but resveratrol supplementation demonstrated controversial results. Chachay and his colleagues [25] found an increment in AST and ALT after supplementing NAFLD patients with 3000 mg of resveratrol, while supplementing 600 mg and 150 mg of resveratrol for 12 weeks did not induce changes in AST, ALT, and GGT levels [26,28]. However, serum levels of AST, GGT, and ALT are general biomarkers for liver damage rather than liver function. Therefore, the factors contributing to their decreased response to polyphenol supplementation could be multifactorial, potentially involving reductions in inflammatory cytokines including TNF-α and IL-6. These cytokines are key mediators of hepatocellular inflammation and cell injury [54].

NAFLD is intricately associated with inflammation, which affects hepatocytes and manifests systemically by elevated levels of CRP in NAFLD patients [55]. Moreover, pro-inflammatory cytokines such as TNF-α, IL-1β, and IL-6 have a crucial function in the progression of NAFLD by stimulating hepatocellular inflammation and inducing fibrosis and cell necrosis [54]. Polyphenols have been reported to ameliorate pro-inflammatory cytokines in NAFLD animal models in parallel with histopathological improvements [11]. In the current systematic review, curcumin, resveratrol, genistein, green tea extract, and hesperidin have effectively reduced pro-inflammatory cytokines. However, studies that examined silybin or silymarin did not report any significant changes in TNF-α, IL-6, CRP, and IL-1β. As mentioned earlier, the anti-inflammatory properties of the polyphenols could be due to the downregulation of pro-inflammatory cytokine expression by inhibiting the phosphorylation of NF-κB and MAPK [56]. Activating IL-1β and TNF-α requires the COX-2 enzyme, which is found to be inhibited by curcumin, resveratrol, green tea extract, and hesperidin [57,58,59,60]. Dysregulation of lipid metabolism stimulates endoplasmic reticulum stress, which further amplifies the inflammatory response in hepatic cells by upregulating NF-κB [56,61]. Resveratrol, curcumin and green tea have been found to regulate fatty acid oxidation which further leads to lower levels of inflammatory cytokines [62,63,64].

The accumulation of lipid metabolites in hepatocytes exceeding 5% is a histological criterion for diagnosing NAFLD, associated with the induction of oxidative stress under NAFLD pathophysiology. According to the included trials, turmeric, curcumin, green tea extract, hesperidin, and silymarin significantly decreased TG, LDL, and TC [14,15,16,20,33,34,42]. The primary transcription factors responsible for lipids accumulation and metabolism are PPAR-α, PPAR-γ, SREBP-1c, and C/EBP-α gene [61]. Regulating lipid metabolism via PPAR-γ and SREBP-1c pathways attenuated hepatic steatosis in an NAFLD rat model after supplementation with curcumin, turmeric, green tea extract, hesperidin, and silymarin [65]. While resveratrol downregulated the gene expression of PPAR-γ and SREBP-1c in the NAFLD rat model [66], the current reviewed RCTs did not show consistent decrease in lipid profile. However, activating AMPK, a potent inhibitor for SREBP-1c, through reducing de novo lipogenesis has become a therapeutic target for addressing NAFLD. Also, AMPK plays another role in ameliorating NAFLD by inhibiting the phosphorylation of acetyl-CoA carboxylase, preventing fatty acid synthesis, and increasing fatty acid oxidation. Turmeric, curcumin, resveratrol, green tea extracts, silybin, and genistein have activated AMPK in rats and cell models [67]. These studies have also found that AMPK activation is coupled with a decrement in insulin resistance, which is also reflected by improvements in fasting blood glucose and, ultimately, in NAFLD scores [67]. Due to polyphenol supplementation, insulin resistance showed a significant improvement, reflected by a reduction in BMI and body weight among NAFLD patients based on the RCTs. While sirtuins are a family of signaling proteins involved in cellular metabolic regulation, curcumin, turmeric, resveratrol, green tea extract, and silymarin have been found to activate sirtuin, a deacetylate of liver kinase B_1_ (LKBT_1_), followed by AMPK activation [62,63,64,68].

Most recently, several systematic reviews have been performed to determine the role of specific classes of polyphenols in ameliorating NAFLD [3,56]. However, our study has a strength in highlighting certain explanations related to the included studies. The inconsistent findings across all the included studies are due to the different types of polyphenols, which vary in molecular structures and properties [49]. The biological properties of polyphenols are also affected by their interaction with the food matrix, which reduces the bioavailability of polyphenols [69]. Furthermore, the dietary intake of NAFLD patients in the included studies could skew the findings, especially if the trials were carried out in different geographical locations where the polyphenol intake of the population sample could be higher or lower. For example, the Mediterranean diet has been associated with moderate to high polyphenol intake and prevents or reverses NAFLD [70]. Another strength that can be noticed in our study is the attempt to gather all the recent available RCTs across all types of polyphenols. This is crucial because it allows for additional research on the role of phenolic compounds in improving NAFLD.

However, a few limitations are present in the current systematic review. The findings of the included studies had difficulty in generalization, as the RCTs had a small sample population. Because these samples were derived from various countries, their findings might not be generalizable to the global population. The significance of this consideration is particularly apparent when evaluating data sourced from clinical trials performed in developing and developed countries, as baseline differences among the populations might exist. Furthermore, the included studies tended to examine the exact exposure measurement, but they assessed the outcomes differently. Finally, this study investigated RCTs published in English, which might not represent all available evidence and could lead to inconsistent conclusions.

## 5. Conclusions

The current study attempted to provide a systematically comprehensive evaluation of the effect of dietary polyphenol supplementation against NAFLD development. Our analysis of 29 studies reveals that curcumin, silymarin, and hesperidin demonstrate significant potential in ameliorating critical markers of liver dysfunction, including a reduction in liver enzymes (AST, ALT, and GGT), improvement in lipid profile (LDL, HDL, TC, and TG), and reduction in inflammatory cytokines (TNF-α, IL-6, and CRP). However, certain polyphenols (such as resveratrol) have failed to show consistent improvement in NAFLD makers, and other polyphenols (such as naringenin and genistein) lack RCTs. Given the variability in the provided evidence, further high-quality, large-scale RCTs with standardized interventions are still required to initiate clear clinical guidelines for using polyphenol supplementation among NAFLD patients. Until such evidence is available, clinicians should consider the preliminary findings and exercise caution when recommending polyphenol supplements.

## Figures and Tables

**Table 1:** Characteristics of the included studies in the systematic review.

First Author’s Last Name, Year, Country	Sample Size I/C	Mean Age (yrs)	Gender (M/F)	Study Design	Intervention and Dose	Control	Duration	Main Results
Turmeric and Curcumin								
(Kalhori et al. 2022) Iran [14]	I: 21 C: 21	42.5 ± 8.3	I: 10/11 C: 8/13	Randomized, double-blind, placebo-controlled trial	Turmeric 3000 mg	Placebo 3000 mg	12 weeks	↓ BMI ↓ AST ↓ ALT ↓ lipid profile ↓ HOMA-IR ↓ sirtuin-1
(jarhahzadeh et al. 2021), Iran [15]	I: 32 C: 32	42.2 ± 10.5	I: 19/13 C: 19/13	Randomized double-blind clinical trial	Turmeric 2000 mg	Placebo 2000 mg	12 weeks	↓ ALT ↓ AST ↓ lipid profile ↓ GGT
(Mirhafez et al. 2021) Iran [16]	I: 35 C: 37	42.5 ± 5.6	I: 17/11 C: 11/17	Double-blind, randomized, placebo-controlled Trial	Curcumin 250 mg	Placebo 250 mg	8 weeks	↓ BMI ↓ ALT ↓ AST ↓ lipid profile ↓ adverse events
(Saberi-Karimian et al. 2020) Iran [17]	I: 27 C: 28	18–70	NA	Randomized controlled trial	Curcuminoids 500 mg + piperine 5 mg	Placebo	8 weeks	↓ NAFLD severity score ↓ TNF-α ↓ MCP-1 ↓ EGF ↓ ALT ↓ AST
(Cicero et al. 2020) Iran [18]	I: 40 C: 40	55 ± 5.6	NA	Double-blind, placebo-controlled clinical trial	Curcumin 200 mg + phosphatidylserine 120 mg + phosphatidylcholine 480 mg + piperine 8 mg	Placebo	8 weeks	↓ Fasting plasma insulin (FPI), ↓ HOMA index ↓ waist circumference, ↓ blood pressure, ↓ TG ↑ HDL-C, ↓ GGT ↓ fatty liver index ↓ cortisol
(Moradi et al. 2020) Iran [19]	I_1_: 11 I_2_: 11 C: 23	65.8 ± 4.8	I: 0/22 C: 0/22	Randomized double-blind clinical trial	I_1_: Curcumin 80 mg I_2_: resistance training + curcumin 80 mg	Resistance training without curcumin or placebo	12 weeks	↓ ALT ↓ AST ↔ BMI ↔ weight
(Saadati et al. 2019) Iran [20]	I: 27 C: 23	46.8 ± 3.6	I: 13/14 C: 14/9	Randomized, placebo-controlled, clinical trial	Curcumin 1500 mg	Placebo	12 weeks	↓ weight ↓ BMI ↓ waist circumference ↓ Hip circumference ↓ blood lipid profile ↓ HOMA-IR ↓ hepatic steatosis ↓ Hepatic fibrosis ↓ALT ↓ AST ↓ GGT
(Panahi et al. 2019) Iran [21]	I: 35 C: 35	46.4 ± 11.2	I: 20/15 C: 19/16	Randomized controlled parallel-group trial	curcuminoids 500 mg coadministered with 5 mg of piperine	Placebo	12 weeks	↓ ESR ↓ AST ↓ ALT ↓ ALP ↔ TG ↔ HDL-C ↓ LDL ↓ NAFLD severity
(Rahmani et al. 2016) Iran [22]	I: 37 C: 40	47.89 ± 2.5	I: 19/21 C: 19/21	Randomized double-blind placebo-controlled trial	curcumin 70 mg	Placebo	8 weeks	↓ BMI, ↓ TC ↓ LDL-C ↓ TG ↓ ALT ↓ AST ↓ glucose
Resveratrol								
(Heebøll et al. 2016) Denmark [23]	I: 13 C: 13	18–70	I: 9/4 C: 8/5	Placebo-controlled, randomized clinical trial	Resveratrol 1500 mg	Placebo	26 weeks	↔ ALP ↓ AST ↓ ALT ↔ GGT ↔ BMI ↔ Weight ↔ TNF-α ↔ Lipid profile ↔ HOMA-IR
(Chen et al. 2015) China [24]	I: 30 C: 30	45.5 ± 3.8	I: 8/22 C: 20/10	Randomized controlled trial	Resveratrol 300 mg	Placebo	12 weeks	↓ AST ↓ ALT ↔ GGT ↓ HOMA-IR ↓ TC ↓ LDL ↔ TG ↓ TNF-α ↔ BMI ↔ Weight
(Chachay et al. 2014) Australia [25]	I: 10 C: 10	46.8 ± 12.5	I: 10/0 C: 10/0	Randomized, double-blind, placebo-controlled trial	Resveratrol 3000 mg	Placebo	8 weeks	↔ Weight ↔ BMI ↑ HOMA-IR ↑ ALT ↑ AST ↔ TNF-α ↔ blood lipid profile ↔ CRP ↓ IL-6
(Farzin et al. 2020) Iran [26]	I: 25 C: 25	39.5 ± 11.2	NA	Randomized, double-blind, placebo-controlled clinical trial	Resveratrol 600 mg	Placebo	12 weeks	↓ waist circumference ↓ BMI ↓ weight ↔ lipid profile ↔ liver enzymes (AST, ALT, ALP and GGT).
(Faghihzadeh et al. 2014) Iran [27]	I: 25 C: 25	45.4 ± 12.5	I: 18/7 C: 17/8	Randomized, double-blind, controlled clinical trial	Resveratrol 500 mg	Placebo	12 weeks	↔ weight ↔ BMI ↑ GGT ↓ ALT ↓ AST ↔ CRP ↑ IL-6 ↔ TNFα ↓ Fibrosis
(Kantartzis et al. 2018) Denmark [28]	I: 53 C: 52	18–70	I: 22/32 C: 30/24	Randomized, double-blind, placebo-controlled clinical trial	Resveratrol 150 mg	Placebo	12 weeks	↔ NAFLD score ↔ HOMA-IR ↔ AST ↔ ALT ↔ GGT ↔ Hs-CRP ↔ IL-6
Anthocyanins								
(Sangsefidi et al. 2021) Iran [29]	I: 25 C: 25	42.6 ± 10.7	NA	Double-blind randomized clinical trial	Anthocyanins 320 mg (cornelian cherry fruit extract)	caramel color, allura red color, and natural flavorings with a color, appearance, taste, and texture similar to the cornelian cherry extract, but without any anthocyanins.	12 weeks	-
Naringenin								
(Namkhah et al. 2021) Iran [30]	I: 22 C: 22	45.7 ± 10.7	I: 12/10 C: 13/9	Randomized, double-blind, placebo-controlled, clinical trial	Naringenin 200 mg	Placebo	4 weeks	↓ NAFLD score ↓ Weight ↓ BMI ↔ AST ↔ ALT ↓ TG ↓ TC ↓ LDL
Genistein								
(Amanat et al. 2018) Iran [31]	I: 41 C: 41	45.55 ± 6.1	I: 30/11 C: 31/10	Randomized, controlled trial	Genistein 250 mg	Placebo	8 weeks	↓ Weight ↓ BMI ↓ waist circumference ↓ TG ↔ TC ↔ LDL ↔ HDL ↔ AST ↔ ALT ↓ HOMA-IR ↓ TNF-α ↓ IL-6
Catechin								
(Sakata et al. 2013) Iran [32]	I_1_: 5 I_2_: 7 C: 5	30–65	NA	Double-blind placebo-controlled study	I_1_: 200 mg catechin I_2_:1080 mg catechin	green tea-flavored beverage with 0 mg catechin	12 weeks	↔ weight ↔ BMI ↓ ALT
Green tea extract								
(Hussain, 2017) Pakistan [33]	I: 40 C: 40	20–55	I: 26/14 C: 28/12	Randomized placebo-controlled trial	Green tea extract 500 mg	Placebo 500 mg of cellulose	12 weeks	↓ body weight ↓ BMI ↓ HOMA-IR ↓ Lipid profile ↓ AST ↓ ALT ↓ Hs-CRP
Hesperidin								
(Yari et al. 2021) Iran [34]	I_1_: 22 I_2_: 24 I_3_: 22 C_1_: 21	44–47	I_1_:11/11 I_2_: 13.9/10.1 I_3_: 13/12 C:10.5/11.5	Open-labeled randomized controlled trial	I_1_: Hesperidin 1000 mg I_2_: Flaxseed 30,000 mg I_3_: Hesperidin 1000 mg + Flaxseed 30,000 mg	30,000 mg flaxseed or Placebo	12 weeks	↓ BMI ↓ waist-circumference ↓ ALT ↔ AST ↓ GGT ↓ HOMA-IR ↓ Lipid profile ↓ Hs-CRP ↓ TNF-α ↓ NF-κB ↓ Fibrosis score ↓ Steatosis score
(Cheraghpour et al. 2019) Iran [35]	I: 24 C: 25	47.30 ± 12.73	I: 10/13 C: 12/12	Randomized, double-blind, controlled clinical trial	Hesperidin 1000 mg	Placebo	12 weeks	↓ ALT ↔ AST ↓ GGT ↔ HOMA-IR ↓ TG ↓ TC ↔ LDL-C ↓ Hs-CRP ↓ TNF-α ↓ NF-κB ↔ Fibrosis score
Silymarin and Silybin								
(Federico et al. 2019) Italy [36]	I: 60 C: 30	47–54	I: 29/31 C: 19/11	Randomized placebo-controlled trial	Silybin-phospholipid 606 mg, + vitamin D 20 mg + vitamin E 30 mg	Placebo	24 weeks	↓ ALT ↓ GGT ↓ HOMA-IR ↓ Steatosis ↔ lipid profile ↔ CRP ↔ TNF-α ↓ IL-18 ↓ TGF-β
(Loguercio et al. 2012) Italy [37]	I: 69 C: 69	40.8 ± 10.3	NA	Randomized controlled trial	Silybin 94 mg + phosphatidylcholine 194 mg + vitamin E	Placebo	48 weeks	↔ BMI ↓ ALT ↓ AST ↓ GGT ↓ HOMA-IR ↓ NAFLD score
(Wah Kheong, 2017) Malaysia [38]	I: 49 C: 50	49.6 ± 12.7	I: 24/25 C: 22/28	Randomized, double-blind, placebo-controlled trial	silymarin 2100 mg	Placebo	48 weeks	↔ body weight ↔ HOMA-IR ↓ AST ↔ ALT ↔ GGT ↔ TC ↔ HDL ↔ LDL ↓ TG ↓ Fibrosis ↓ NAFLD score
(Navarro et al. 2019) Italy [39]	I_1_: 26 I_2_: 27 C: 25	47–50 ± 11.7	I_1_: 18/9 I_2_: 13/13 C: 14/11	Randomized, double-blind, placebo-controlled trial	I_1_: Silymarin 420 mg I_2_: Silyamarin 700 mg	Placebo	48 weeks	↔ AST ↔ ALT ↔ HOMA-IR ↔ Fibrosis ↔ NAFLD score
(Hashemi, 2009) Iran [40]	I: 50 C: 50	39.5 ± 10.3	I:28/22 C: 29/21	Placebo-controlled trial	Silymarin 280 mg	Placebo	24 weeks	↔ BMI ↔ lipid profile ↓ AST ↓ ALT
(Solhi et al. 2014) Iran [41]	I: 33 C: 31	39.36–43.6	I: 19/14 C: 15.5/15.5	Randomized clinical trial	Silymarin 210 mg	Placebo	8 weeks	↓ AST ↓ ALT
(Anushiravani et al. 2019) Iran [42]	I: 30 C: 30	47.0 ± 9.1	I + C: 77/73	Double-blind randomized placebo-controlled trial	Silymarin 140 mg	Placebo	12 weeks	↓ BMI ↓ waist-circumference ↓ Lipid profile ↓ AST ↓ ALT

I: Intervention group, C: Control group, M: Male, F: Female, NM: Not Mentioned. ↑: significantly increased, ↓: significantly decreased, ↔: no significant change.

## Data Availability

Data related to the current article are available from the corresponding author (seham.alraish@uaeu.ac.ae) upon request due to (complexity and volume of the data extracted from multiple sources).

## Supplementary Materials

The following supporting information can be downloaded at: https://www.mdpi.com/article/10.3390/nu16234150/s1, Table S1: Electronic search strategies. PRISMA 2020 Checklist [71].
